# *In Vitro* antibacterial and antibiotic-potentiation activities of four edible plants against multidrug-resistant gram-negative species

**DOI:** 10.1186/1472-6882-13-190

**Published:** 2013-07-25

**Authors:** Jaurès AK Noumedem, Marius Mihasan, Jules R Kuiate, Marius Stefan, Dumitru Cojocaru, Jean P Dzoyem, Victor Kuete

**Affiliations:** 1 Department of Biochemistry, Faculty of Science, University of Dschang, Dschang, Cameroon; 2 Department of Biochemistry and Molecular Biology, Faculty of Biology, University ALI Cuza, Iasi, Romania

**Keywords:** Antibacterial activities, Edible plants, Gram-negative bacteria, Multidrug resistance, Efflux pumps

## Abstract

**Background:**

The present study was designed to investigate the antibacterial activities of the methanol extracts of four Cameroonian edible plants, locally used to treat microbial infections, and their synergistic effects with antibiotics against a panel of twenty nine Gram-negative bacteria including Multi-drug resistant (MDR) phenotypes expressing active efflux pumps.

**Methods:**

The broth microdilution method was used to determine the minimum inhibitory concentrations (MICs) of the extracts [alone and in the presence of the efflux pumps inhibitor (EPI) Phenylalanine-Arginine *β-*Naphtylamide (PAβN)], and those of antibiotics in association with the two of the most active ones, *Piper nigrum* and *Telfairia occidentalis*. The preliminary phytochemical screening of the extracts was conducted according to the standard phytochemical methods.

**Results:**

Phytochemical analysis showed the presence of alkaloids and flavonoids in all studied extracts. Other chemical classes of secondary metabolites were selectively present in the extracts. The results of the MIC determination indicated that the crude extracts from *P. nigrum* and *V. amygdalina* were able to inhibit the growth of all the twenty nine studied bacteria within a concentration range of 32 to 1024 μg/mL. At a similar concentration range (32 to 1024 μg/mL) the extract from *T. occidentalis* inhibited the growth of 93.1% of the tested microorganisms. At MIC/2 and MIC/5, synergistic effects were noted between the extracts from *P. nigrum* and *T. occidentalis* and seven of the tested antibiotics on more than 70% of the tested bacteria.

**Conclusion:**

The overall results of the present study provide information for the possible use of the studied edible plants extracts in the control of bacterial infections including MDR phenotypes.

## Background

Despite the impressive scientific progress in vaccination and chemotherapy, infectious diseases remain a serious health issue. Following the massive and inappropriate use of antibiotics, bacteria have developed various mechanism of resistance; consequently, infectious diseases remain one of the leading causes of morbidity worldwide [1]. Microbial infections constitute a major public health problem in developing countries [2] where the high cost of antibiotics makes them unaffordable to the majority of the population. Therefore, the discovery of new antimicrobial agents is still relevant nowadays. Among the bacterial resistance mechanisms, efflux of antibiotics plays an important role; In fact it is widely recognized that the expression of efflux pumps encoded by house-keeping genes in bacteria is largely responsible for the phenomenon of intrinsic antibiotic resistance [3]. Also, the shortcomings of the drugs available today and the scarcity of novel antibiotics propel the discovery of new chemotherapeutic agents from medicinal plants [4]. The medicinal properties of many phytochemicals have been demonstrated [5]. In addition, promising new concepts such as the efflux pump inhibitors [6,7], and synergy between antibiotics and phytochemicals are now been explored.

The present work was therefore designed to investigate the antibacterial potential of four Cameroonian edible plants used traditionally in the treatment of bacterial infections, namely the fruits of *Piper nigrum* L (Piperaceae), the leaves of *Telfairia occidentalis* Hook. F. (Cucurbitaceae) and *Vernonia amygdalina* Del. (Asteraceae) and the fruits of *Syzygium aromaticum* [L.] Merr & Perry (Myrtaceae) against MDR bacteria expressing active efflux *via* the Resistance-Nodulation Cell Division (RND)-type pumps.

## Methods

### Plant material and extraction

The four edible plants used in this work were purchased from Dschang local market, West Region of Cameroon in June 2010. The collected plants material were the fruits of *Piper nigrum*, the fruits of *Syzygium aromaticum*, the leaves of *Telfairia occidentalis* and the leaves of *Vernonia amygdalina*. These plants were identified by M. Victor Nana of the National Herbarium (Yaounde-Cameroon) where all the voucher specimens were available under the reference numbers (see Table 1). The air dried and powdered sample (1 kg) from each plant was extracted with methanol (MeOH) for 48 h at room temperature. The extracts were then filtered and concentrated under reduced pressure to give the crude extracts. All extracts were kept at 4°C until further investigations.

**Table 1:** Plants used in the present study and evidence of their bioactivities

**Plant (family); and voucher number**^**a**^	**Traditional uses**	**Parts used**	**Bioactive or potentially bioactive components**	**Bioactivities of extracts and/or compounds**
*Piper nigrum* L. (Piperaceae); 25818/SFRcam	Cardiovascular diseases, intoxication, inflammation, bacterial, fungal and parasitic infections, respiratory diseases, asthma [8]	Seeds, bark, leaves	Piperine, pipene [9], piperamides, piperamine [10], pellitorine [11]	Anti-apoptotic [12,13], antibacterial [8,14], antidepressant [15] antifungal [16], analgesic, anti-inflammatory [17], antidiarrhoeal [18], antimutagenic, antioxidative, increase plasma [19], antipyretic [17], immuno-modulatory, antispasmodic [20,21], asthma, obesity, sinus antispermatogenic, antithyroid, antitumor Ciprofloxacin potentiator, transcription inhibitor, insecticidal, hepatoprotective, increase pancreatic enzymes, Cytochrome Inhibitor [8]
*Syzygium aromaticum* (Myrtaceae) 28524/HNC	Aphrodisiac, used to treat male sexual disorders [22,23], anti-inflammatory, bacterial infections [24], microbial infections [25,26]	fruits	Eugenol (2-methoxy-4-(2-propenyl) phenol), glycosides, flavonoids, saponins and tannins [23], essential oils	Antipyretic, antispasmodic [27], anticarcinogenic [28], inhibition of 5-Lox enzyme activity in human polymorphonuclear leukocytes cells [29], antioxidant, protection against peroxynitrite-mediated tyrosine nitration and lipid peroxidation [29], antifungal activity of essential oil [26] and antimicrobial [25], antibacterial [30]
*Telfairia occidentalis (*Curcubitaceae); 33423/HNC	Microbial infections, cholesterolemia, liver problems and impaired defense immune systems [31]	Leaves, seeds, roots	Phenols, alkaloids and tannins [32]	Antimicrobial, antioxidant and free radical-scavenger [32,33], antiplasmodial, cure lactating properties, hypoglycemic and antidiabetic [31]
*Vernonia amygdalina* Del. *(*Asteraceae ); 31149/SRFK	Microbial infections [34], hiccups, kidney and stomach problems, discomfort [35], stomach-ache and gastrointestinal infections, malarial fever , cough remedy [36], anti-malarial, purgative, anti-parasitic, eczema blood glucose levels control [37], treatment of eczema [37]	Leaves, roots	Flavonoids, saponins and alkaloids [36], vernodalin, vernomygdin, vernonioside B1 and vernoniol B1 [38]	Active anticancer [39], antimalarial and antiparasitic agents [40], Hypoglycaemic [41], antimicrobial, antibacterial [35,40,42], antihelminthic, anti-shitosomal, tumor inhibitor [38], hypolipidaemic and antioxidant properties [43]

^a^(HNC): Cameroon National Herbarium; (SRFC): *Société des réserves forestières du Cameroun*.

### Preliminary phytochemical investigations

The major classes of secondary metabolites such as alkaloids, anthocyanins, anthraquinones, flavonoids, phenols, saponins, tannins, sterols and triterpenes were screened according to the common phytochemical methods described by Harbone [44].

### Bacterial strains and culture media

The studied microorganisms included the reference (from the American Type Culture Collection) and clinical (Laboratory collection) strains of *Providencia stuartii, Pseudomonas aeruginosa, K. pneumoniae, Escherichia coli, Enterobacter aerogenes* and *Enterobacter cloacae* (See supporting information Additional file 1: Table S1 for their features). They were maintained in a Nutrient Broth at 4°C and activated on a fresh appropriate Mueller Hinton Agar plates 24 h prior to antimicrobial test. The Mueller Hinton Broth (MHB) was also used for all the antibacterial assays.

### Chemicals for antimicrobial assays

Tetracycline (TET), cefepime (FEP), cloxacillin (CLX), streptomycine (STR), ciprofloxacine (CIP), norfloxacine (NOR), chloramphenicol (CHL), cloxacillin (CLX), ampicillin (AMP), erythromycin (ERY), kanamycin (KAN) and streptomycin (STR) (Sigma-Aldrich, St Quentin Fallavier, France) were used as reference antibiotics. *p*-Iodonitrotetrazolium chloride (INT) and Phenylalanine Arginine *β*-naphthylamide (PAβN) were used as microbial growth indicator and efflux pumps inhibitor (EPI) respectively.

### Bacterial susceptibility determination

The MICs were determined using the rapid INT colorimetric assay [45,46]. Briefly, the test samples were first dissolved in DMSO/MHB. The solution obtained was then added to MHB, and serially diluted two fold (in a 96- wells microplate). One hundred microlitres (100 μL) of inoculum (1.5 × 10^6^ CFU/mL) prepared in MHB was then added. The plates were covered with a sterile plate sealer, then agitated to mix the contents of the wells using a shaker and incubated at 37°C for 18 h. The final concentration of DMSO was set at 2.5% (a concentration at which DMSO does not affect the microbial growth). Wells containing MHB, 100 μL of inoculum and DMSO at a final concentration of 2.5% served as negative control (this internal control was systematically added). Chloramphenicol was used as reference antibiotic. The MICs of samples were detected after 18 h incubation at 37°C, following addition of 40 μL of a 0.2 mg/mL INT solution and incubation at 37°C for 30 minutes. Viable reduce the yellow dye to pink. MIC was defined as the lowest sample concentration that exhibited complete inhibition of microbial growth and then prevented this change [47].

Samples were tested alone and the best three extracts (those from the seeds of *P. nigrum*, *T. occidentalis* and *V. amygdalina*) were also tested in the presence of PAβN at 30 mg/L final concentration. After a preliminary assay on one of the MDR bacteria, *P. aeruginosa* PA124 (See supporting information Additional file 1: Table S2), the two best extracts were those from *P. nigrum* and *T. occidentalis*. They were then selected and tested at MIC/2 and MIC/5 in association with antibiotics. Fractional inhibitory concentration (FIC) was calculated as the ratio of MIC_Antibiotic in combination_/MIC_Antibiotic alone_ and the results were discussed as follows: synergy (*≤* 0.5), indifferent (0.5 to 4), or antagonism (*>* 4) [48,49]. All assays were performed in triplicate.

## Results

### Phytochemical composition and antibacterial activity of the extracts

The results of the qualitative phytochemical analysis showed that each of the tested plant extract contains at least 3 classes of secondary metabolites (Table 2). The antibacterial activities of the extracts alone and in some cases in combination with PAβN on a panel of 29 Gram-negative bacteria are depicted in Table 3. It appears that extracts from *P. nigrum* and *V. amygdalina* inhibited the growth of all the twenty nine tested bacterial strains within a concentration range from 32 to 1024 μg/mL. A good spectrum of antibacterial activity was also recorded with the extract of *T. occidentalis,* its inhibitory effects being observed against 27/29 (93.1%) of the tested microorganisms. The lowest MIC value (32 μg/mL) was obtained with the extract of *P. nigrum* on *P. aeruginosa* PA01.

**Table 2:** Extraction yields, aspects and phytochemical composition of the plant extracts

**Scientific names**	**Part used**	Yield* **(%)**	**Physical aspect**	**Secondary metabolites**								
				**Alkaloids**	**Phenols**	**Tannins**	**Triterpenes**	**Steroids**	**Flavonoids**	**Anthraquinones**	**Anthocyanins**	**Saponins**
*Piper nigrum*	Fruits	13.18	Brown sticky paste	+	+	+	-	-	+	+	-	-
*Syzygium aromaticum*	Fruits	9.49	Dark brown paste	+	-	-	-	-	+	-	-	-
*Telfairia occidentalis*	Leaves	11.58	Brown sticky paste	+	+	+	-	-	+	-	+	-
*Vernonia amygdalina*	Leaves	7.16	Green dark paste	+	+	+	-	+	+	-	-	+

(+): Present; (−): Absent; *the yield was calculated as the ratio of the mass of the obtained methanol extract/mass of the plant powder.

**Table 3:** Minimal Inhibitory Concentration (MIC) in μg/mL of methanol extracts from the studied plants and chloramphenicol

**Bacteria strains**	**Plant extracts and MICs (μg/mL)**				
	***Piper nigrum***	***Syzygium aromaticum***	***Telfairia occidentalis***	***Vernonia amygdalina***	**Chloramphenicol**
*E. coli*					
ATCC8739	128	1024	512	512	4
ATCC10536	256	-	512	512	4
W3110	256	512	256	512	8
MC4100	256	1024	512	512	16
AG100A	128 (64)	-	512 (512)	512 (512)	< 2 (< 2)
AG100Atet	256 (256)	-	512 (512)	1024 (1024)	64 (< 2)
AG102	512 (128)	-	1024 (1024)	256 (256)	64
AG100	256 (32)	-	512 (512)	512 (32)	8 (< 2)
*E. aerogenes*					
ATCC13048	512	1024	512	512	8
EA294	512	-	512	1024	16
CM64	512 (256)	1024	256 (64)	256 (32)	256 (8)
EA3	512 (256)	-	1024 (1024)	512 (512)	256 (128)
EA298	256 (128)	-	512 (512)	256 (256)	64 (< 2)
EA27	512 (256)	-	-	1024 (1024)	≥ 256 (< 2)
EA289	512 (256)	1024	512 (256)	256 (256)	≥ 256 (64)
*K. pneumoniae*					
ATCC11296	256	1024	1024	512	8
KP55	256 (64)	-	512 (512)	512 (512)	32 (4)
KP63	1024 (256)	-	256 (256)	1024 (1024)	64 (< 2)
K2	512	-	-	1024	32
K24	512	-	512	512	16
*P. aeruginosa*					
PA01	32	-	512	512	16
PA124	256 (128)	512	512 (512)	1024 (1024)	32 (< 2)
*P. stuartii*					
ATCC29916	128	-	128	256	16
NAE16	512	1024	512	256	64
PS2636	256	-	512	512	32
PS299645	1024 (1024)	-	1024	512	32
*E. cloacae*		-			
BM47	128	-	512	1024	≥ 256
ECCI69	128	-	512	512	≥ 256
BM67	128 (32)	-	512 (16)	1024 (1024)	128 (32)

(−) MIC > 1024 μg/mL.

### Role of efflux pumps in the susceptibility of Gram-negative bacteria to the tested plant extracts

Fourteen of the studied MDR bacteria were also tested for their susceptibility to the most active plant extracts (*P. nigrum*, *V. amygdalina* and *T. occidentalis*) in the presence PAβN at 30 μg/mL. When combined with extracts, PAβN improved the activity (decrease of MIC values) of *P. nigrum* on almost all of the tested MDR strains [13/14 (92.9%)]. The EPI also improved the activity of *T. occidentalis* against *E. aerogenes* CM64, EA 289 and *E. cloacae* BM67 as well as that of *V. amygdalina* against *E. coli* AG100 and *E. aerogenes* EA 289 (Table 3).

### Effect of the association of extracts with antibiotics

A preliminary study (See supporting information; Additional file 1: Table S2) was performed against *P. aeruginosa* PA124 using the three most active plant extracts. The results permitted the selection of the extracts from *P. nigrum* and *T. occidentalis* with the appropriate sub-inhibitory concentrations of MIC/2 and MIC/5 for further studies. Therefore, the extracts from *P. nigrum* and *T. occidentalis* were combined with eleven antibiotics [TET, DOX, CIP, NOR, STR, KAN, CHL, ERY, FEP, CLX and AMP] separately to evaluate their possible synergistic effects. As results, synergistic effects were observed with the two extracts and most of the tested antibiotics except ß-lactams (AMP, FEP and CLX) (Tables 4 and 5). At MIC/2 and MIC/5 of the extract from *T. occidentalis*, synergistic effects were observed with 7 of the 11 antibiotics (TET, DOX, CIP, NFX, KAN, CHL, ERY) against the tested MDR bacteria (Table 5).

**Table 4:** **MIC of different antibiotics after the association of the extract of *Piper nigrum *at MIC/2, MIC/5 against eleven MDR bacteria strains**

	**Bacterial strains, MIC (μg/mL ) of antibiotics in the absence and presence of the extract**												
**Antibiotics**	**Extract concentration**	**PA124**	**AG100**	**AG102**	**AG100Atet**	**CM64**	**EA3**	**EA27**	**EA289**	**BM67**	**KP55**	**NEA16**	**PBSS (%)**
**TET**	0	8	16	256	64	8	16	64	16	8	> 256	4	
	MIC/2	4(2)^S^	16(1)^I^	≤ 2(≥ 128)^S^	16(4)^S^	≤ 2(> 4)^S^	4(4)^S^	16(4)^S^	16(1)^I^	8(1)^I^	> 256	≤2(> 2)^S^	63.63
	MIC/5	4(2)^S^	16(1)^I^	≤ 2(≥ 128)^S^	16(4)^S^	≤ 2(> 4)^S^	4(4)^S^	8(8)^S^	16(1)^I^	8(1)^I^	> 256	≤ 2(> 2)^S^	63.63
**DOX**	0	16	8	32	32	32	32	32	32	8	32	64	na
	MIC/2	8(2)^S^	≤ 2(> 4)^S^	≤ 2(> 16)^S^	≤ 2(> 16)^S^	≤ 2(> 16)^S^	4(8)^S^	8(4)^S^	32(1)^I^	≤ 2(> 4)^S^	32(1)^I^	4(16)^S^	81.81
	MIC/5	8(2)^S^	≤ 2(> 4)^S^	≤ 2(> 16)^S^	≤ 2(> 16)^S^	4(8)^S^	≤ 2(> 16)^S^	8(4)^S^	16(1)^I^	≤ 2(> 4)^S^	32(1)^I^	≤ 2(> 32)^S^	81.81
**CIP**	0	32	4	128	64	64	128	≤ 2	8	128	64	128	na
	MIC/2	32(1)^I^	≤ 2(> 2)^S^	16(8)^S^	4(16)^S^	8(8)^S^	64(2)^S^	≤ 2	4(2)^S^	64(2)^S^	≤ 2(> 32)^S^	64(2)^S^	90
	MIC/5	32(1)^I^	≤ 2(> 2)^S^	64(2)^S^	16(4)^S^	4(16)^S^	64(2)^S^	≤ 2	4(2)^S^	128(1)^I^	≤ 2(> 32)^S^	128(1)^I^	70
**NFX**	0	128	128	64	128	≤ 2	128	32	64	128	128	256	na
	MIC/2	64(2)^S^	4(32)^S^	≤2(> 32)^S^	16(8)^S^	≤ 2	32(4)^S^	8(4)^S^	16(4)^S^	128(1)^I^	16(8)^S^	128(2)^S^	90
	MIC/5	128(1)^I^	≤ 2(> 64)^S^	64(1)^I^	16(8)^S^	≤ 2	32(4)^S^	16(2)^S^	16(4)^S^	128(1)^I^	16(8)^S^	128(2)^S^	80
**STR**	0	256	≤ 2	256	64	8	32	16	64	≤ 2	4	64	na
	MIC/2	256(1)^I^	≤ 2	64(4)^S^	≤ 2(> 32)^S^	≤ 2(> 4)^S^	≤ 2(> 16)^S^	32(0.5)^I^	4(16)^S^	≤ 2	≤ 2(> 2)^S^	≤ 2(> 32)^S^	77.78
	MIC/5	256(1)^I^	≤ 2	64(4)^S^	≤ 2(> 32)^S^	≤ 2(> 4)^S^	4(8)^S^	16(1)^I^	4(16)^S^	≤ 2	≤ 2(> 2)^S^	≤ 2(> 32)^S^	77.78
**KAN**	0	ND	8	128	32	≤2	16	16	32	≤ 2	8	≤ 2	na
	MIC/2	ND	≤ 2(> 4)^S^	≤ 2(> 64)^S^	16(2)^S^	≤2	≤ 2(> 8)^S^	4(4)^S^	4(8)^S^	≤ 2	16(0.5)	≤ 2	75
	MIC/5	ND	≤ 2(> 4)^S^	≤ 2(> 64)^S^	4(8)^S^	≤2	≤ 2(> 8)^S^	4(4)^S^	4(8)^S^	≤ 2	8(1)^I^	≤ 2	75
**CHL**	0	32	64	> 256	64	256	32	> 256	> 256	64	16	128	na
	MIC/2	16(2)^S^	4(16)^S^	16(> 16)^S^	64(1)^I^	32(8)^S^	4(8)^S^	32(> 8)^S^	> 256	32(2)^S^	8(2)^S^	8(16)^S^	81.81
	MIC/5	32(1)^I^	16(4)^S^	16(> 16)^S^	64(1)^I^	128(2)^S^	4(8)^S^	64(> 4)^S^	> 256	32(2)^S^	16	128(1)^I^	54.54
**ERY**	0	128	32	256	128	> 256	256	64	256	256	256	256	na
	MIC/2	128(1) ^I^	8(4)^S^	64(4)^S^	256(0.5)^I^	32(> 8)^S^	64(4)^S^	32(2)^S^	256(1)^I^	64(4)^S^	16(16)^S^	64(4)^S^	72.72
	MIC/5	128(1)^I^	16(2)^S^	32(4)^S^	128(1)^I^	8(> 32)^S^	256(1)^I^	8(8)^S^	256(1)^I^	128(2)^S^	16(16)^S^	64(4)^S^	63.63
**AMP**	0	128	128	> 256	> 256	> 256	> 256	> 256	>256	> 256	> 256	> 256	na
	MIC/2	128(1)^I^	128(1)^I^	> 256	> 256	> 256	> 256	> 256	> 256	> 256	> 256	> 256	na
	MIC/5	128(1)^I^	128(1)^I^	> 256	> 256	> 256	> 256	> 256	> 256	> 256	> 256	> 256	na
**FEP**	0	> 256	> 256	> 256	> 256	> 256	> 256	> 256	> 256	> 256	> 256	> 256	na
	MIC/2	> 256	> 256	> 256	> 256	> 256	> 256	> 256	> 256	> 256	> 256	> 256	na
	MIC/5	> 256	> 256	> 256	> 256	> 256	> 256	> 256	>256	> 256	> 256	> 256	na
**CLX**	0	> 256	> 256	> 256	> 256	> 256	> 256	> 256	> 256	> 256	> 256	> 256	na
	MIC/2	> 256	16(> 16)^S^	> 256	> 256	> 256	> 256	> 256	> 256	> 256	> 256	> 256	1.1
	MIC/5	> 256	32(> 8)^S^	> 256	> 256	64(> 4)^S^	> 256	> 256	> 256	> 256	> 256	> 256	18.2

^a^Antibotics [*TET* : tetracycline, *DOX*: doxycyclin, *CIP* : ciprofloxacin, *NOR* : norfloxacin, *STR* : streptomycin, *KAN* : kanamycin, *CHL*: chloramphenicol, *ERY* : erythromycin, *AMP* : ampicillin, *FEP*: cefepime, *CLX* : cloxacillin]. ^b^Bacterial strains: *Escherichia coli* [AG100, AG100A, AG100Atet], *Pseudomonas aeruginosa* [PA124], *Enterobacter aerogenes* [CM64, EA3, EA27, EA289], *Enterobacter cloacae* [BM67], *Klebsiella pneumoniae* [KP55], *Providencia stuartii* [NAE16]. ^c^PBSS: percentage of bacteria strain on which synergism has been observed; (): fold increase in MIC values of the antibiotics after association with plants extract; S: Synergy, I: Indifference; na: not applicable.

**Table 5:** **MIC of different antibiotics after the association of the extract of *Telfairia occidentalis *at MIC/2, MIC/5 against eleven MDR bacteria**

**Antibiotics**^**a**^	**Bacterial strains**^**b**^**, MIC (μg/mL ) of antibiotics in the absence and presence of the extract**												**PBSS**^**c **^**(%)**
	**Extract concentration**	**PA124**	**AG100**	**AG102**	**AG100Atet**	**CM64**	**EA3**	**EA27**	**EA289**	**BM67**	**KP55**	**NEA16**	
TET	0	8	16	256	64	8	16	64	16	8	> 256	4	
	MIC/2	4(2)^S^	8(2)^S^	256(1)^I^	32(2)^S^	≤ 2(> 4)^S^	≤ 2(> 8)^S^	64(1)^I^	8(2)^S^	4(2)^S^	256(> 1)^s^	≤ 2(> 2)^S^	81.8
	MIC/5	4(2)^S^	8(2)^S^	256(1)^I^	32(2)^S^	≤ 2(> 4)^S^	≤ 2(> 8)^S^	64(1)^I^	8(2)^S^	4(2)^S^	256(> 1)^s^	4(1)^I^	72.7
DOX	0	16	8	32	32	32	32	32	32	8	32	64	na
	MIC/2	2(8)^S^	≤ 2(> 4)^S^	16(2)^S^	4(8)^S^	4(8)^S^	≤ 2(> 16)^S^	4(8)^S^	16(2)^S^	4(2)^S^	≤ 2(> 16)^S^	32(2)^S^	100
	MIC/5	8(2)^S^	≤ 2(> 4)^S^	32(1)^I^	8(4)^S^	≤ 2(> 16)^S^	≤ 2(> 16)^S^	8(4)^S^	16(2)^S^	≤ 2(> 4)^S^	64(< 0.5)	64(1)^I^	72.7
CIP	0	32	4	128	64	64	128	≤ 2	8	128	64	128	na
	MIC/2	32(1)^I^	≤ 2(> 2)^S^	64(2)^S^	≤ 2(> 32)^S^	16(4)^S^	≤ 2(> 64)^S^	≤ 2	≤ 2(> 4)^S^	128(1)^I^	≤ 2(> 32)^S^	128(1)^I^	70
	MIC/5	32(1)^I^	≤ 2(> 2)^S^	64(2)^S^	≤ 2(> 32)^S^	16(4)^S^	≤ 2(> 64)^S^	≤ 2	≤ 2(> 4)^S^	128(1)^I^	≤ 2(> 32)^S^	128(1)^I^	70
NFX	0	128	128	64	128	≤ 2	128	32	64	128	128	256	na
	MIC/2	64(2)^s^	4(32)^S^	32(2)^S^	4(32)^S^	≤ 2	4(32)^S^	4(8)^S^	32(2)^S^	128(1)^I^	≤ 2(> 64)^S^	128(2)^S^	90
	MIC/5	32(4)^s^	4(32)^S^	32(2)^S^	4(32)^S^	≤ 2	4(32)^S^	4(8)^S^	16(4)^S^	128(1)^I^	8(16)^S^	256(1)^I^	80
STR	0	256	≤ 2	256	64	8	32	16	64	≤ 2	4	64	na
	MIC/2	256(1)^I^	≤ 2	256(1)^I^	16(4)^S^	≤ 2(> 4)^S^	≤ 2(> 16)^S^	16(1)^I^	≤ 2(> 32)^S^	≤ 2	≤ 2(> 2)^S^	16(4)^S^	66.7
	MIC/5	256(1)^I^	≤ 2	256(1)^I^	8(8)^S^	≤ 2(> 4)^S^	≤ 2(> 16)^S^	16(1)^I^	≤ 2(> 32)^S^	≤ 2	≤ 2(> 2)^S^	16(4)^S^	66.7
KAN	0	ND	8	128	32	≤ 2	16	16	32	≤ 2	8	≤ 2	na
	MIC/2	ND	4(2)^S^	128(1)^I^	8(4)^S^	≤ 2	≤ 2(> 8)^S^	≤ 2(> 8)^S^	≤ 2(> 16)^S^	≤ 2	8(1)^I^	≤ 2	71.4
	MIC/5	ND	4(2)^S^	128 (1)^I^	≤ 2(> 16)^S^	≤ 2	≤ 2(> 8)^S^	≤ 2(> 8)^S^	≤ 2(> 16)^S^	≤ 2	8(1)^I^	≤ 2	71.4
CHL	0	32	64	> 256	64	256	32	> 256	64	16	128	128	na
	MIC/2	16(2)^s^	32(2)^S^	> 256	64(1)^I^	≤ 2(> 128)^S^	4(8)^S^	256(> 1)^s^	16(4)^S^	≤ 2(> 8)^S^	16(8)^S^	16(8)^S^	90
	MIC/5	32(1)^I^	32(2)^S^	> 256	64(1)^I^	≤ 2(> 128)^S^	8(4)^S^	256(> 1)^s^	32(2)^S^	16(1)^I^	64(2)^S^	64(2)^S^	70
ERY	0	128	32	256	128	> 256	256	64	256	256	256	256	na
	MIC/2	64(2)^s^	16(2)^S^	> 256	32(4)^S^	≤ 2	4(64)^S^	32(2)^S^	64(4)^S^	32(8)^S^	8(32)^S^	128(2)^S^	72.7
	MIC/5	64(2)^s^	32(1)^I^	> 256	32(4)^S^	64(> 4)^S^	4(64)^s^	64(1)^I^	256(1)^I^	32(8)^S^	16(16)^S^	128(2)^S^	45.5
AMP	0	128	128	> 256	> 256	> 256	> 256	> 256	> 256	> 256	> 256	> 256	na
	MIC/2	256(0.5) ^I^	128(1)^I^	> 256	> 256	> 256	256(> 1)^s^	> 256	> 256	> 256	> 256	> 256	na
	MIC/5	256(0.5) ^I^	128(1)^I^	> 256	> 256	> 256	> 256	> 256	> 256	> 256	> 256	> 256	na
FEP	0	ND	> 256	> 256	> 256	> 256	> 256	> 256	> 256	256	> 256	> 256	na
	MIC/2	ND	> 256	> 256	> 256	> 256	> 256	> 256	> 256	> 256(< 1)	8(> 32)^S^	> 256	1.1
	MIC/5	ND	> 256	> 256	> 256	> 256	> 256	> 256	> 256	> 256(< 1)	> 256	> 256	na
CLX	0	> 256	256	> 256	> 256	> 256	> 256	> 256	> 256	> 256	> 256	> 256	na
	MIC/2	> 256	64(> 4)^S^	> 256	> 256	256(≥2) ^S^	64(> 4)^S^	> 256	> 256	> 256	> 256	> 256	27.3
	MIC/5	> 256	64(> 4)^S^	> 256	> 256	256(≥2) ^S^	128(> 2)^S^	> 256	> 256	> 256	> 256	> 256	27.3

^a^Antibotics [*TET* : tetracycline, *DOX*: doxycyclin, *CIP*: ciprofloxacin, *NOR* : norfloxacin, *STR* : streptomycin, *KAN* : kanamycin, *CHL*: chloramphenicol, *ERY* : erythromycin, *AMP* : ampicillin, *FEP*: cefepime, *CLX*: cloxacillin]. ^b^Bacterial strains: *Escherichia coli* [AG100, AG100A, AG100Atet], *Pseudomonas aeruginosa* [PA124], *Enterobacter aerogenes* [CM64, EA3, EA27, EA289], *Enterobacter cloacae* [BM67], *Klebsiella pneumoniae* [KP55], *Providencia stuartii* [NAE16]. ^c^PBSS: percentage of bacteria strain on which synergism has been observed; (): fold increase in MIC values of the antibiotics after association with plants extract; S: Synergy, I: Indifference; na: not applicable tract; S: Synergy, I: Indifference; na: not applicable.

## Discussion

### Antibacterial activities and chemical composition of the tested extracts

Many secondary metabolites belonging to alkaloids, anthocyanins, anthraquinons, flavonoids, phenols, saponins, sterols, tannins and triterpenes were detected in the tested plant extracts. Several compounds from the investigated classes of phytochemicals were reported for their antibacterial activities [50,51], and their presence in the tested extracts could explain their antibacterial effects. The differences in bacterial susceptibility to the extracts may be either due to the differences in cell wall composition and/or genetic content of their plasmids [52] or to the differences in the composition and the mechanism of action of the bioactive compounds [53]. As shown in Table 3, the three most active plants (*P. nigrum, T. occidentalis* and *V. amygdalina*) possess more classes of phytochemicals than the extract from *S. aromaticum*. Each of the three most active plant extracts contains at least four classes of secondary metabolites namely alkaloids, phenols, flavonoids and tannins. However, it should be noted that the activity does not depend on the number of classes of detected bioactive compounds, but mostly on their concentration. The inhibitory activity of *P. nigrum* was previously reported against some bacteria such as *Staphylococcus aureus*, *Bacillus cereus*, *Streptococcus faecalis*, *Pseudomonas aeruginosa*, *Salmonella typhi* and *Escherichia coli*[54], and the data reported in this study confirms the anti-infective potential of this plant. It has also been demonstrated that the acetone-ethanol extract of the leaves from *V. amygdalina* was weakly active against *K*. *pneumoniae, E. coli, S. aureus*, *B. cereus, S. dysentriae* and *S. typhimurium*[35] with MIC values ranged from 7.5 mg/mL to 25 mg/mL [42]. These activities are in accordance with the results obtained in the present work, but we observed higher antibacterial activity of this plant on all 29 bacteria including MDR phenotypes (with MIC values ranging between 256 and 1024 μg/mL).

### Role of efflux pumps in the susceptibility of Gram-negative bacteria to the tested extracts and effects of the association of some extracts with antibiotics

All the bacterial strains tested with a combination of plant extract and PAβN were proven to possess multidrug resistance efflux pumps [55-59]. Tripartite efflux systems, mainly those clinically described such as AcrAB-TolC in Enterobacteriaceae or MexAB-OprM in *P. aeruginosa* play a central role in multidrug resistance of pathogenic Gram-negative bacteria [55,56]. PAßN, a potent inhibitor of the RND efflux systems is especially active on AcrAB-TolC and MexAB-OprM [57,58] and does not present any intrinsic effect on the bacteria at the concentration of 30 μg/mL used in this work [59]. In the presence of PAßN at this concentration, significant increase of the activity of the extract from *P. nigrum* was noted against 13/14 of the tested MDR bacteria. This shows that at least one active compound from this plant, acting inside the bacteria cell could be the substrate of efflux pumps. From this observation, it can be suggested that the association of the extract of *P. nigrum* and efflux pump inhibitors could be helpful in the fight against infections due to MDR bacteria [5].

Moreover, we demonstrated in this study that the beneficial effect of the combination of two of the tested plant extracts namely those from *P. nigrum* and *T. occidentalis,* with the first line antibiotics could be achieved. Their synergistic effects with antibiotics were noted on more than 70% of the tested MDR bacteria (with seven antibiotics), also suggesting that some of their constituents can act as efflux pump inhibitor [49]. This hypothesis is emphasized by the fact that these extracts were more synergistic with antibiotics acting inside the bacteria cells. Besides, it has already been proved that the extract from *P. nigrum* can also act by improving the penetration of antibiotics in cells *via* membrane alteration [54]. However, further phytochemical investigations will be done to isolate the active constituents of *P. nigrum*, *T. occidentalis* and *V. amygdalina*. Besides, toxicological studies will be carried out to evaluate their safety.

## Conclusion

The overall results of the present study provide baseline information for the possible use of the tested plants and mostly *P. nigrum, T. occidentalis and V. amygdalina* in the control of infections due to MDR Gram-negative bacteria. In addition, the extracts from *P. nigrum* and *T. occidentalis* could be used in association with antibiotics to combat multidrug resistant pathogens.

## Abbreviations

AMP: Ampicillin; ATCC: American type culture collection; CEF: Cefepime; CFU: Colony forming unit; CHL: Chloramphenicol; CIP: Ciprofloxacin; DMSO: Dimethylsulfoxyde; EPI: Efflux pump inhibitor; ERY: Erythromycin; FIC: Fractional inhibitory concentration; INT: p-iodonitrotetrazolium chloride; KAN: Kanamycin; MDR: Multidrug resistant; MHB: Mueller hinton broth; MIC: Minimal inhibitory concentration; NOR: Norfloxacin; PAßN: Phenylalanine arginine *ß*-Naphthylamide; RND: Resistance nodulation-cell division; STR: Streptomycin; TET: Tetracycline

## Competing interest

The authors declare that there are no conflict of interest.

## Authors’ contributions

JAKN*,* MM and MS carried out the study; VK designed the experiments. JAKN, MM, JPD and VK wrote the manuscript; VK, JRK and DC supervised the work; VK provided the bacterial strains; all authors read and approved the final manuscript.

## Pre-publication history

The pre-publication history for this paper can be accessed here:

http://www.biomedcentral.com/1472-6882/13/190/prepub

## Supplementary Material

Additional file 1 Table S1 Bacterial strains and features, **Table S2.** Effects of different concentrations of extracts on the MIC (μg/mL) of antibiotics against the PA124 strain. **Table S3.** FIC of different antibiotics after the association of the extracts of black *Piper nigrum* fruits and *Telfairia occidentalis* leaves at the concentrations MIC/2, MIC/5 against eleven actives efflux pumps MDR bacteria strains. Click here for file
