# Supplementary material for: In Vitro antibacterial and antibiotic-potentiation activities of four edible plants against multidrug-resistant gram-negative species
Source: BMC Complement Altern Med. 2013 Jul 25;13:190. doi: 10.1186/1472-6882-13-190 (PMC3734210; doi:10.1186/1472-6882-13-190)
Supplement: Additional file 1 Table S1 — Bacterial strains and features, Table S2. Effects of different concentrations of extracts on the MIC (μg/mL) of antibiotics against the PA124 strain. Table S3. FIC of different antibiotics after the association of the extracts of black Piper nigrum fruits and Telfairia occidentalis leaves at the concentrations MIC/2, MIC/5 against eleven actives efflux pumps MDR bacteria strains. [file 1472-6882-13-190-S1.doc]

***In Vitro* Antibacterial and Antibiotic-potentiation Activities of four Edible Plants against Multidrug-resistant Gram-negative Species.**

Jaurès A.K. Noumedem^a,b^, Marius Mihasan^b^, Jules R. Kuiate^a*^, Marius Stefan^b^, Jean P. Dzoyem^a^, Dumitru Cojocaru^b^ and Victor Kuete^a**^

*^a^Department of Biochemistry, Faculty of Science, University of Dschang, Cameroon*

*^b^Department of Biochemistry and Molecular Biology, Faculty of Biology, University ALI. Cuza, Iasi, Romania*

Correspondence to: ** [*kuetevictor@yahoo.fr*](mailto:kuetevictor@yahoo.fr) *(V.Kuete); *jrkuiate@yahoo.com (J.R. Kuiate)*

**[See Main Manuscript]**

**Table S1. Bacterial strains and features**

| **Strains** | **Features** | **References** |
| --- | --- | --- |
| ***Escherichia coli*** |  |  |
| ATCC8739 and ATCC10536 | Reference strains |  |
| AG100 | Wild-type *E. coli* K-12 | [1] |
| AG100A | AG100 *ΔacrAB*::KAN^R^ | [1, 2] |
| AG100A_TET_ | Δ*acrAB* mutant AG100, owing *acrF* gene markedly over-expressed; TET^R^ | [1] |
| AG102 | Δ*acrAB* mutant AG100 | [3] |
| MC4100 | Wild type *E. coli* |  |
| W3110 | Wild type *E. coli* | [4, 5] |
| ***Enterobacter aerogenes*** |  |  |
| ATCC13048 | Reference strains |  |
| EA-CM64 | CHL^R^ resistant variant obtained from ATCC13048 over-expressing the AcrAB pump | [6] |
| EA3 | Clinical MDR isolate; CHL^R^, NOR^R^, OFX^R^, SPX^R^, MOX^R^, CFT^R^, ATM^R^, FEP^R^ | [7] |
| EA27 | Clinical MDR isolate exhibiting energy-dependent norfloxacin and chloramphenicol efflux with KAN^R^ AMP^R^ NAL^R^ STR^R^ TET^R^ | [7] |
| EA289 | KAN sensitive derivative of EA27 | [8] |
| EA298 | EA 289 *tolC::*KAN^R^ | [8] |
| EA294 | EA 289 *ΔacrAB*: *::*KAN^R^ | [8] |
| ***Enterobacter cloacae*** |  |  |
| ECCI69 | Clinical isolates | Laboratory collection of UNR-MD1, University of Marseille, France |
| BM47 | Clinical isolates | Laboratory collection of UNR-MD1, University of Marseille, France |
| BM67 | Clinical isolates | Laboratory collection of UNR-MD1, University of Marseille, France |
| ***Klebsiella pneumoniae*** |  |  |
| ATCC12296 | Reference strains |  |
| KP55 | Clinical MDR isolate, TET^R^ , AMP^R^, ATM^R^, CEF^R^ | [9] |
| KP63 | Clinical MDR isolate, TET^R^, CHL^R^, AMP^R^, ATM^R^ | [9] |
| K24 | AcrAB-TolC | Laboratory collection of UNR-MD1, University of Marseille, France |
| K2 | AcrAB-TolC | Laboratory collection of UNR-MD1, University of Marseille, France |
| ***Providencia stuartii*** |  | **[10]** |
| NEA16 | Clinical MDR isolate, AcrAB-TolC |
| ATCC29914 | Clinical MDR isolate, AcrAB-TolC |
| PS2636 | Clinical MDR isolate, AcrAB-TolC |
| PS299645 | Clinical MDR isolate, AcrAB-TolC |
| ***Pseudemonas aeruginosa*** |  |  |
| PA 01 | Reference strains |  |
| PA 124 | MDR clinical isolate | [11] |

^a^AMP, ATM^R^, CEF^R^, CFT^R^, CHL^R^, FEP^R^, KAN^R^, MOX^R^, STR^R^, TET^R^. Resistance to ampicillin, aztreonam, cephalothin, cefadroxil, chloramphenicol, cefepime, kanamycin, moxalactam, streptomycin, and tetracycline; MDR : Multidrug resistant.

**Table S2.** Effects of different concentrations of extracts on the MIC (µg/ml) of antibiotics against the PA124 strain

|  | **Tested plants, concentrations and MIC of antibiotics in** µg/ml | | | | | | | | | | | |
| --- | --- | --- | --- | --- | --- | --- | --- | --- | --- | --- | --- | --- |
|  | ***Piper nigrum*** | | | | ***Telfairia occidentalis*** | | | | ***Vernonia amygdalina*** | | | |
|  | 0 | CMI/2 | CMI/5 | CMI/10 | 0 | CMI/2 | CMI/5 | CMI/10 | 0 | CMI/2 | CMI/5 | CMI/10 |
| **TET** | 8 | 4 | 4 | 4 | 8 | 4 | 4 | 8 | 8 | 8 | 8 | 8 |
| **DOX** | 16 | 8 | 8 | 8 | 16 | 2 | 8 | 8 | 16 | 16 | 16 | 16 |
| **CIP** | 32 | 32 | 32 | 32 | 32 | 32 | 32 | 32 | 32 | 32 | 32 | 32 |
| **NFX** | 128 | 64 | 128 | 128 | 128 | 16 | 32 | 32 | 128 | 128 | 128 | 128 |
| **STR** | 256 | 256 | 256 | 256 | 256 | 256 | 256 | 256 | 256 | 256 | 256 | 256 |
| **KAN** | ND | ND | ND | ND | ND | ND | ND | ND | ND | ND | ND | ND |
| **CHL** | 32 | 16 | 32 | 32 | 32 | 16 | 32 | 32 | 32 | 32 | 32 | 32 |
| **ERY** | 128 | 128 | 128 | 128 | 128 | 64 | 64 | 128 | 128 | 128 | 128 | 128 |
| **BAC** | 256 | 256 | 256 | 256 | 256 | 256 | 256 | 256 | 256 | 128 | 256 | 256 |
| **AMP** | 128 | 128 | 128 | 128 | 128 | 128 | 128 | 128 | 128 | 128 | 128 | 128 |
| **FEP** | ND | ND | ND | ND | ND | ND | ND | ND | ND | ND | ND | ND |
| **CLX** | >256 | >256 | >256 | >256 | >256 | >256 | >256 | >256 | >256 | >256 | >256 | >256 |

**TET : tetracycline, DOX: doxycyclin, CIP : ciprofloxacin, NOR : norfloxacin, STR : streptomycin, KAN : kanamycin, CHL: chloramphenicol, ERY : erythromycin, AMP : ampicillin, FEP: cefepime,; CLX : cloxacillin.**

Table S3. FIC of different antibiotics after the association of the extracts of black *Piper nigrum* fruits and *Telfairia* *occidentalis* leaves at the concentrations MIC/2, MIC/5 against eleven actives efflux pumps MDR bacteria strains.

|  |  | ***Associated plants, bacterial strains and FIC values*** | | | | | | | | | | | | | | | | | | | | | |
| --- | --- | --- | --- | --- | --- | --- | --- | --- | --- | --- | --- | --- | --- | --- | --- | --- | --- | --- | --- | --- | --- | --- | --- |
|  |  | ***P. nigrum*** | | | | | | | | | | | ***T. occidentalis*** | | | | | | | | | | |
|  |  | **Bacteria strains** | | | | | | | | | | | **Bacteria strains** | | | | | | | | | | |
| Antibio-tics | Extract Concentra-tions | PA124 | AG100 | AG102 | AG100ATet | C M64 | EA3 | EA27 | EA289 | B M67 | KP55 | NEA16 | PA124 | AG100 | AG102 | AG100ATet | CM64 | EA3 | EA27 | EA289 | B M67 | KP55 | NEA16 |
| TET | CMI/2 | 2 | 1 | ≤0.008 | 0.25 | ≤0.25 | 0.25 | 0.25 | 1 | 1 | ND | ≤0.5 | 0.5 | 1 | 0.25 | 0.125 | ≤0.25 | ≤0.125 | ≤0.031 | 0.25 | 0.25 | ND | 1 |
|  | CMI /5 | 0.5 | 1 | ≤0.008 | 0.25 | ≤0.25 | 0.25 | 0.125 | 1 | 1 | ND | ≤0.5 | 0.5 | 1 | 0.5 | 0.125 | ≤0.25 | ≤0.125 | ≤0.031 | ≤0.125 | ≤0.125 | ≤0.5 | 1 |
| DOX | CMI/2 | 0.5 | ≤0.25 | ≤0.063 | ≤0.063 | ≤0.063 | 0.125 | 0.25 | 1 | ≤0.25 | 1 | 0.125 | 0.5 | ≤0.25 | 0.125 | 0.25 | 0.5 | 0.063 | 0.5 | 0.25 | ≤0.25 | 1 | 0.25 |
|  | CMI /5 | 0.5 | ≤0.25 | ≤0.063 | ≤0.063 | 0.125 | ≤0.063 | 0.25 | 0.5 | ≤0.25 | 1 | ≤0.031 | 0.5 | 0.5 | 0.25 | 1 | 0.125 | ≤0.063 | 0.5 | 1 | ≤0.25 | 1 | 0.5 |
| CIP | CMI/2 | 1 | ≤0.5 | 0.125 | 0.25 | 0.125 | 0.5 | ND | 0.5 | 0.5 | ≤0.031 | 0.5 | 0.5 | ≤0.5 | 0.25 | 0.063 | ≤0.031 | 0.5 | ND | ≤0.25 | 1 | ≤0.031 | 0.5 |
|  | CMI /5 | 1 | ≤0.5 | 0.5 | 0.25 | 0.063 | 0.5 | ND | 0.5 | 1 | ≤0.031 | 1 | 1 | ≤0.5 | 0.25 | 0.063 | 0.063 | 0.5 | ND | ≤0.25 | 1 | ≤0.031 | 1 |
| NFX | CMI/2 | 0.5 | 0.031 | ≤0.031 | 0.125 | ND | 0.25 | 0.25 | 0.25 | 1 | 0.125 | 0.5 | 0.5 | 0.25 | 0.125 | 0.125 | ≥0.5 | ≤0.031 | 0.5 | ≤0.031 | 0.25 | 0.031 | 1 |
|  | CMI /5 | 1 | ≤0.016 | 0.063 | 0.25 | ND | 0.25 | 0.5 | 0.25 | 1 | 0.125 | 0.5 | 0.25 | 0.25 | 0.25 | 0.25 | ND | ≤0.016 | 0.5 | ≤0.031 | 1 | 0.031 | 1 |
| STR | CMI/2 | 1 | ND | 0.5 | 0.031 | ≤0.25 | 0.063 | 2 | 0.063 | ND | ≤0.5 | `≤0.031 | 1 | ND | 0.5 | 0.125 | ≤0.25 | 0.5 | ≤0.125 | ≤0.031 | ND | ≤0.5 | ≤0.031 |
|  | CMI /5 | 1 | ND | 0.25 | ≤0.031 | 0.25 | 0.125 | 1 | 0.063 | ND | ≤0.5 | ≤0.031 | 1 | ≥2 | ≥2 | 1 | ≤0.25 | 0.5 | ≤0.125 | ≤0.031 | ND | ≤0.5 | ≤0.031 |
| KAN | CMI/2 | ND | ≤0.25 | ≤0.016 | 0.5 | ND | ≤0.125 | 0.25 | 0.125 | ND | 4 |  | ND | 0.5 | 0.25 | 0.25 | ND | ≤0.125 | 1 | 0.25 | ND | 1 | ND |
|  | CMI /5 | ND | ≤0.25 | ≤0.016 | 0.5 | ND | ≤0.125 | 0.25 | 0.125 | ND | 1 | ND | ND | ≤0.031 | 0.125 | 1 | ND | ≤0.125 | 1 | ≤0.063 | ND | 0.5 | ND |
| CHL | CMI/2 | 0.5 | 0.25 | ≤0.063 | 1 | 0.25 | 0.125 | ≤0.125 | ND | 0.5 | 0.5 | 0.063 | 0.5 | 0.25 | ≤0.063 | 0.25 | ≤0.008 | 0.5 | 0.031 | 0.063 | 0.125 | 2 | 0.063 |
|  | CMI /5 | 1 | 0.25 | ≤0.031 | 1 | 0.5 | 0.125 | ≤0.125 | ≤0.5 | 0.5 | ≤0.125 | ≤0.016 | 1 | 0.25 | ≤0.031 | 0.5 | ≤0.008 | 0.5 | ≤0.016 | ≤0.063 | 0.25 | 2 | 0.063 |
| ERY | CMI/2 | 0.5 | 0.5 | 0.125 | 1 | ≤0.016 | 1 | 0.25 | 1 | 0.5 | 0.063 | 0.25 | 0.5 | 0.5 | 0.063 | 0.125 | ≤0.004 | ≤0.008 | 0.25 | 0.5 | 0.5 | 0.125 | 0.5 |
|  | CMI /5 | 1 | 0.25 | 0.25 | 2 | ≤0.063 | 0.25 | 0.5 | 1 | 0.25 | 0.063 | 0.25 | 0.5 | 0.5 | 0.125 | 1 | 1 | ≤0.004 | ≤0.008 | 1 | 0.5 | 0.5 | 0.5 |
| BAC | CMI/2 | 1 | ND | ND | ND | ND | ND | ND | 0.25 | ND | ND | ND | 1 | ND | 0.5 | 0.125 | ≤0.25 | 0.5 | ≤0.125 | ≤0.031 | ND | ≤0.5 | ND |
|  | CMI /5 | 1 | ND | ND | ND | ND | ND | ND | 0.25 | ≤0.5 | ND | ND | 1 | ND | ≤0.5 | ND | ND | ND | ND | 1 | ≤0.016 | ≤0.125 | ND |
| AMP | CMI/2 | ≥4 | 1 | ND | ND | ND | ND | ND | ND | ND | ND | ND | ND | 0.5 | 0.25 | 0,25 | ND | ≤0.125 | 1 | 0.25 | ND | 1 | ≤0.063 |
|  | CMI /5 | ND | ND | ND | ND | ND | ND | ND | ND | ND | ND | ND | ≥4 | 0.5 | ≤0.125 | ≤0.125 | ND | ND | ≤0.125 | ND | ≤0.125 | ≤0.031 | ≤0.063 |
| FEP | CMI/2 | ND | ND | ND | ND | ND | ND | ND | ND | ND | ND | ND | ND | 0.031 | ND | ≤0.25 | ND | ND | ≤0.031 | ≤0.25 | ND | ND | ND |
|  | CMI /5 | ND | ND | ND | ND | ND | ND | ND | ND | ND | ≥0.5 | ND | ND | ND | ND | ND | ≤0.25 | ND | ND | ND | 1 | ND | ND |
| CLX | CMI /5 | ND | 0.063 | ND | ND | ≤0.125 | ND | ND | ND | ND | ND | ND | ND | ≥2 | ND | ≤0.25 | ND | ND | ND | ND | ND | ND | ND |
|  | CMI /5 | ND | 0.125 | ND | ND | ≤0.125 | ND | ND | ND | ND | ND | ND | ND | ≥2 | ND | ≤0.25 | ND | ND | ND | ND | ND | ND | ND |

**TET : tetracycline, DOX: doxycyclin, CIP : ciprofloxacin, NOR : norfloxacin, STR : streptomycin, KAN : kanamycin, CHL: chloramphenicol, ERY : erythromycin, AMP : ampicillin, FEP: cefepime, CLX : cloxacillin.**

**References**
